# Phytochemical Analysis and Establishment of Embryogenic Cell Suspension and *Agrobacterium*-mediated Transformation for Farmer Preferred Cultivars of West African Plantain (*Musa* spp.)

**DOI:** 10.3390/plants9060789

**Published:** 2020-06-24

**Authors:** Temitope Jekayinoluwa, Jaindra Nath Tripathi, George Obiero, Edward Muge, Leena Tripathi

**Affiliations:** 1 International Institute of Tropical Agriculture (IITA), 30709-00100 Nairobi, Kenya; T.Jekayinoluwa@cgiar.org (T.J.); j.tripathi@cgiar.org (J.N.T.); 2 Centre for Biotechnology and Bioinformatics, University of Nairobi, 30197-00100 Nairobi, Kenya; George.obiero@uonbi.ac.ke; 3 Department of Biochemistry, University of Nairobi, 30197-00100 Nairobi, Kenya; mugeek@uonbi.ac.ke

**Keywords:** banana, plantain, somatic embryogenesis, cell suspension, phytochemicals, *Agrobacterium*-mediated transformation

## Abstract

Banana and plantain are among the foremost staple food crops providing food and livelihood to over 500 million people in tropical countries. Despite the importance, their production is hampered due to several biotic and abiotic stresses. Plant tissue culture techniques such as somatic embryogenesis and genetic transformation offer a valuable tool for genetic improvement. Identification and quantification of phytochemicals found in banana and plantain are essential in optimizing in vitro activities for crop improvement. Total antioxidants, phenolics, flavonoids, and tannins were quantified in various explants obtained from the field, as well as in vitro plants of banana and plantain cultivars. The result showed genotypic variation in the phytochemicals of selected cultivars. The embryogenic cell suspensions were developed for three farmer-preferred plantain cultivars, Agbagba, Obino l’Ewai, and Orishele, using different MS and B5-based culture media. Both culture media supported the development of friable embryogenic calli (FEC), while MS culture media supported the proliferation of fine cell suspension in liquid culture media. The percentage of FEC generated for Agbagba, Obino l’Ewai, and Orishele were 22 ± 24%, 13 ± 28%, and 9 ± 16%, respectively. Cell suspensions produced from FECs were successfully transformed by *Agrobacterium*-mediated transformation with reporter gene constructs and regenerated into whole plants.

## 1. Introduction

Banana and plantain (*Musa* spp.) belonging to family *Musaceae* are an important staple food and cash crops in tropical and sub-tropical countries [1]. They originated from Southeast Asia; however, West and Central Africa is considered as a secondary hub of diversification for plantain with over 100 cultivars [2]. The cultivated varieties of banana and plantain are derived from either *Musa acuminata* (A genome) or/and *Musa balbisiana* (B genome) as well as their hybrids [3]. The various types of banana and plantain can be categorized as AA and AB (diploids), and AAA, AAB, and ABB (triploids), based on their genomic constitution. The plantains are triploid hybrids of *Musa acuminata* and *Musa balbisiana* having AAB genome [1]. Plantains are further divided into four types (False horn, French horn, Horn, and French) based on the morphology of the inflorescence [1,4]. Horn and False horn type of plantains have a high market value because of their desirable bunch characteristics, while French type is more valuable to breeders because it is fertile [5]. 

Plantains are mainly grown in West and Central Africa, Latin-America, and Asia by smallholder farmers [1]. In West Africa, it is a valuable food crop consumed as a major staple. For instance, in Nigeria, it is the third staple food crop after cassava and yams [6]. It is consumed either as a snack, main meal, or a meal accompaniment at any time of the day. In Nigeria and Cameroon, an average of about 265 g and 402 g, respectively, of plantain is consumed by an individual per day [7]. This crop is grown all year-round, contributing to its market value especially to smallholder farmers, who depend primarily on it as a livelihood. False horn type of plantain cultivars Agbagba and Orishele, and French type cultivar Obino l’Ewai are important cultivars grown by smallholder farmers in Nigeria for local consumption [8,9]. These plantain cultivars are included in the breeding program to develop improved varieties with resistance to major diseases like black Sigatoka and pests such as weevils and nematodes [10]. 

Even though plantain produces fruits throughout the year, the major harvest comes in the dry season from December to March, when most other starchy staples are in short supply or difficult to harvest [11]. Therefore, it is considered as a crucial crop, which bridges the hunger gap during the dry season [2]. In addition to its several advantages and uses, plantain biomass is a potential source of natural fiber that needs to be explored as a feasible alternative to reduce the dependency on cotton as a sole raw material for textile industries [12].

Several pests and diseases threaten the production of banana and plantain [1]. This impact is felt more by subsistence farmers, who are more vulnerable to the losses associated with the continuous spread of the disease [1]. At times, varieties resistant to some of these economically significant diseases are not available. The importance of the crop for food and raw material highlights the need to develop the disease and pest resistant varieties preferred by the local farmers to meet the demand in a growing population. 

Biotechnological approaches like somatic embryogenesis (SE) is a reliable tissue culture tool that is important for plant genetic modification and trait improvement, mass propagation of plant stock, production of synthetic seeds, and callus for developing cell suspension [13]. It also facilitates the manipulation associated with plant cell complexity at the cellular level, which involves regenerating a plant from the somatic embryos. This remodeling process of the somatic embryos can either occur naturally [14] or artificially induced through the use of synthetic plant growth regulators (PGR) [15]. Direct somatic embryogenesis is a process of inducing somatic embryos on the surface or cut ends of plant tissue without the callus stage while indirect somatic embryogenesis involves the development of somatic embryos through callus [16]. The embryogenic potential of banana and plantain is highly influenced by age, type, and physiology of explants, culture media, type of PGR, stoichiometry between endogenous and exogenous PGR, and other stress-related factors such as temperature and pH [17,18]. The phytochemicals or secondary metabolites, as well as other cellular and molecular programs such as methylation and transcriptional factors, could also influence somatic embryogenesis in banana and plantains as reported in other crops [19,20,21]. 

Banana and plantain produce several phytochemicals that occur at different explants and growth stages. These phytochemicals do not only influence the developmental stages of SE features and regeneration into complete plantlets but also have useful applications in the pharmaceutical industries and the production of bio-pesticides [22,23,24,25]. Phenolic compounds, flavonoids, tannins, and antioxidants are some of the primary phytochemicals in banana and plantain [26]. Some of these compounds could promote SE development and germination of somatic embryos [27,28,29]. 

The type and concentration of phytochemicals required to confer positive response on a plant tissue are difficult to determine because the influences that could trigger the production of phytochemicals is genotype-dependent. Besides, metabolic products of secondary metabolites, it may inhibit, support, or have non-lethal effects in establishing in vitro culture and SE [30,31]. Availability of a broad-spectrum of these chemicals and its identification could serve as a basis for understanding plant response under certain conditions, improving crop genetic material [32], and targeted strategy for handling and scaling-up plant tissue culture procedures for optimal output. 

Crop improvement through genetic transformation has gained wide applications in plant genetics [33]; it comes in handy, especially when there is no gene in crop germplasm that could confer resistance to a specific pest or disease. The genetic transformation employs the use of the physical method, biological method, or a combination of both. The physical techniques, including particle bombardment and electroporation and biological methods such as *Agrobacterium tumefaciens* and *Agrobacterium rhizogenes* mediated transformation, have been extensively used for genetic modification of crops [34,35,36]. Among these methods, *Agrobacterium*-mediated transformation is the most preferred method for genetically modifying most of the crops. *Agrobacterium*-mediated transformation systems are reported for banana using different type of explants such as embryogenic cell suspension (ECS) [37,38], apical meristems [39] and intercalary meristematic tissues [40]. The most preferred explant for the transformation of banana and plantain is ECS; however, the generation of ECS is variety-dependent, labor-intensive, and lengthy [41]. Although protocols for *Agrobacterium*-mediated transformations using ECS are available for *Musa* spp., they are genotype-dependent and targeted at specific varieties [42]. 

This study sought to develop procedures for the development of ECS, and *Agrobacterium*-mediated transformation of farmer preferred cultivars, Agbagba, Orishele, and Obino l’Ewai, of West African plantain. These cultivars are common landraces grown in West Africa, and their improvement would have a positive impact on farmers as well as their market value. The study focused on determining the antioxidant capacity, total phenolics, flavonoids, and tannins present in the banana and plantain while optimizing procedures for the development of ECS and *Agrobacterium*-mediated transformation for farmer preferred cultivars of West African plantain. 

## 2. Results and Discussion

As the preferred explant for genetic transformation of plantain is embryogenic cell suspensions (ECS), here, we optimized the protocol to generate ECS of three West African farmers preferred cultivars of plantain using B5 and MS-based media. The phytochemicals were quantified in various banana and plantain cultivars, which can provide information for the manipulation of the crop. Further, the transformation capacity of the ECS generated was determined by the use of an optimized *Agrobacterium*-mediated approach to facilitate the incorporation of a transgene into the plantain cell. 

### 2.1. In Vitro Shoot Initiation and Proliferation of Plantain Cultivars

Following initiation of the in vitro cultures of plantain cultivars via meristem culture in proliferation medium containing Murashige and Skoog medium (MS) salts and vitamins, 10 mg/L ascorbic acid, 4 mg/L BAP, 0.18 mg/L IAA, 30 g/L sucrose, 3 g/L gelrite, pH: 5.8 at 26 ± 2 °C, 38 µmol/m^2^/s 16/8 h photoperiod, the cultures were sub-cultured every four weeks. All the three plantain cultivars tested showed the ability to regenerate to the whole plant from shoot tip cultures. The micropropagation or proliferation potential among the plantain cultivars varied significantly with the type of cultivar and subculture number (*p* < 0.001) (Table 1). There was no significant variation for the mean number of shoots per explant among the three cultivars of plantain at first subculture (S1). However, Orishele had the highest mean shoot value of 2.5 ± 0.7 at S1. At S2 and S3, the mean number of shoots for the three cultivars varied significantly (*p* < 0.001). The highest mean number of shoots was observed for Agbagba at various subculture cycles with 29.4 ± 8.2 shoots per explant at S2 and 119 ± 42.2 shoots per explant at S3. However, least number of shoots was found for Obino l’Ewai with 20.4 ± 11.5 and 49.4 ± 25.9 shoots per explant at S2 and S3, respectively. Similar reports indicated that banana and plantain varieties respond differently to in vitro micropropagation [43,44]. Although the plantain cultivars tested are of the same genome (AAB), they exhibited varying proliferation potentials. The difference in the number of shoots might be related to the higher concentration of total antioxidants, phenolics and flavonoid content in in vitro plantlets of Agbagba compared to Obino l’Ewai and Orishele.

### 2.2. Quantification of Targeted Phytochemicals in Banana and Plantain Cultivars

**Figure 1:**
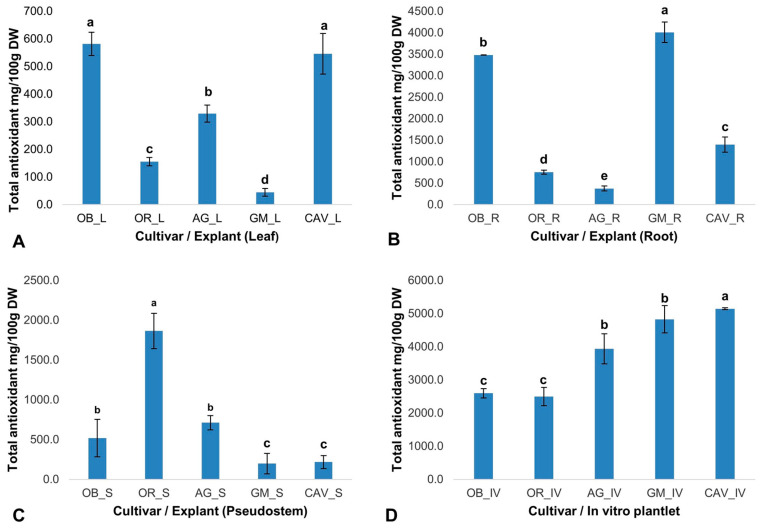
Total antioxidant activity in various explants of field-grown plants and in vitro plantlets of different cultivars of banana and plantain. (**A**) Leaf (L) explant; (**B**) Root (R) explant; (**C**) Pseudostem (S) explant; (**D**) In vitro (IV) plantlet. Each bar represents the mean of triplicate samples (*n* ≥ 3), and the error line indicates the standard deviation. Means with a different letter (a–e) significantly from each other at *p* ≤ 0.05. OB—Obino l’Ewai; OR—Orishele; AG—Agbagba; GM—Gonja Manjaya; CAV—Cavendish Williams; DW—Dry weight of sample.

The phytochemicals play a significant role in in vitro regeneration and manipulations. Therefore, the presence and quantity of targeted phytochemicals (total antioxidants, total phenolics, flavonoids, and tannins) were determined on selected farmer preferred cultivars of banana and plantain, namely Cavendish Williams, Orishele, Agbagba, Obino l’Ewai, and Gonja Manjaya. The cultivars and explant types determine the level of phytochemicals variation with no influence on genome differences. In general, in vitro samples in the absence of exogenous antioxidant and plant growth hormone gave the highest antioxidant potential in Cavendish Williams (5142 ± 28 mg/100 g dry weight), Gonja Manjaya (4828 ± 413 mg/100 g dry weight), Agbagba (3935 ± 450 mg/100 g dry weight), Obino l’Ewai (2595 ± 137 mg/100 g dry weight) and Orishele (2499 ± 276 mg/100 g dry weight) compared to explants collected from field-grown plants except for root explant of Obino l’Ewai, where the total antioxidant activity was higher compared to that in vitro plantlets (Figure 1). A similar trend was observed in the total phenolic content of these cultivars suggesting the influence of micropropagation procedures on increased production of biochemicals. Similar results were reported in blueberry, where subjecting lowbush blueberry to tissue culture procedure resulted in an increased phenolic content and antioxidant activity [45]. 

#### 2.2.1. Total Antioxidant Activity

The free radical scavenging activity of the total antioxidants varied across the explants and cultivars tested. Gonja Manjaya recorded the highest antioxidant activity (4010 ± 236 mg/100 g dry weight) in root and lowest in the leaf explant (44 ± 14 mg/100 g dry weight) (Figure 1). For in vitro samples, the highest antioxidant activity to reduce 2, 2-diphenyl-1-picrylhydrazyl (DPPH) to Diphenylpicrylhydrazine (DPPH-H) was in Cavendish Williams (5142 ± 28 mg/100 g dry weight) and lowest in Orishele (2595 ± 137 mg/100 g dry weight). The antioxidant potential in Orishele was about half the concentration found in Cavendish Williams. On the other hand, substantial antioxidant capacity for Orishele was found in the pseudostem (1864 ± 222 mg/100 g dry weight). Agbagba produced the least antioxidant activity in the root (375 ± 60 mg/100 g dry weight) (Figure 1). 

#### 2.2.2. Phenolic Content

**Figure 2:**
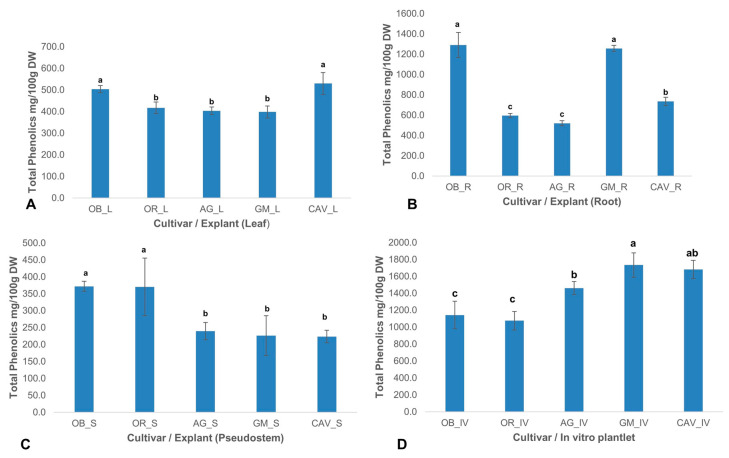
Total phenolics in various explants of field-grown plants and in vitro plantlets of different cultivars of banana and plantain. (**A**) Leaf (L) explant; (**B**) Root (R) explant; (**C**) Pseudostem (S) explant; (**D**) In vitro (IV) plantlet. Data are presented as means of triplicate samples (*n* ≥ 3) and standard deviation. Means with different letters (a—c) are significantly different from each other at *p* ≤ 0.0001 (highly significant). OB—Obino l’Ewai; OR—Orishele; AG—Agbagba; GM—Gonja Manjaya; CAV—Cavendish Williams; DW—Dry weight of sample.

There was a significant (*p* < 0.0001) difference in the level of total phenolics among different cultivars and explants used in the study. The highest levels of phenolic content were observed in in vitro samples for Gonja Manjaya, followed by Cavendish Williams, Agbagba, and lowest in Obino l’Ewai, and Orishele (Figure 2).

#### 2.2.3. Flavonoid Content

**Figure 3:**
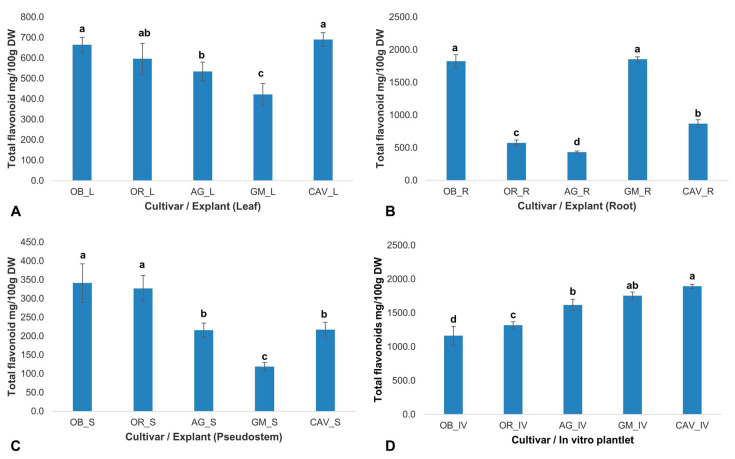
Total flavonoids in explants of field-grown plants and in vitro plantlets of different cultivars of banana and plantain. (**A**) Leaf (L) explant; (**B**) Root (R) explant; (**C**) Pseudostem (S) explant; (**D**) In vitro (IV) plantlet. Each bar represents the mean of triplicate samples (*n* ≥ 3), and the error line indicates the standard deviation. Means with different letters (a—d) are significantly different from each other at *p* ≤ 0.001 (highly significant). OB—Obino l’Ewai; OR—Orishele; AG—Agbagba; GM—Gonja Manjaya; CAV—Cavendish Williams; DW—Dry weight of sample.

The concentration of flavonoids varied significantly (*p* ≤ 0.001) across different cultivars and explants used in the study. The highest concentration of flavonoids was in the root of Gonja Manjaya (1855 ± 37 mg/100 g dry weight), while among in vitro samples, the highest and lowest concentrations were in Cavendish Williams (1895 ± 29 mg/100 g dry weight), and Obino l’Ewai (1163 ± 140 mg/100 g dry weight), respectively (Figure 3). The root samples of Agbagba yielded the lowest concentration of 434 ± 19 mg/100 g dry weight. 

#### 2.2.4. Tannin Content

**Figure 4:**
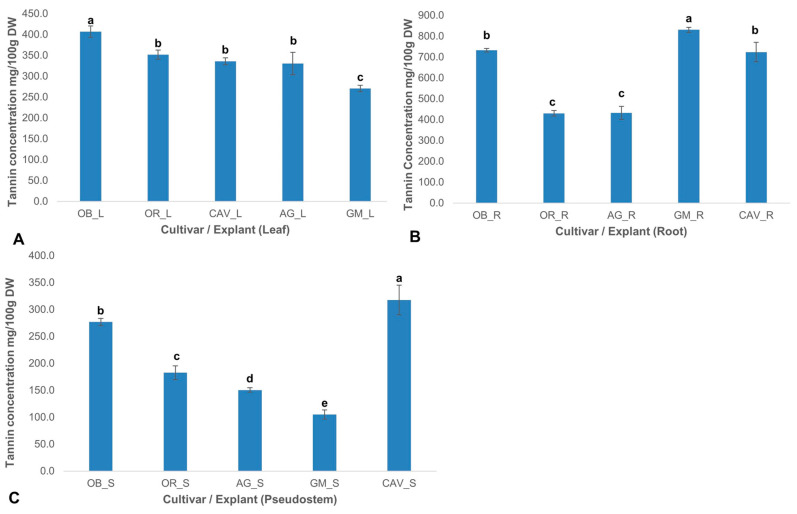
Total tannins in explants of field-grown plants of different cultivars of banana and plantain. (**A**) Leaf (L) explant; (**B**) Root (R) explant; (**C**) Pseudostem (S) explant. Data are presented as means of triplicate samples (*n* ≥ 3) and standard deviation. Means with a different letter (a–e) differ significantly from each other at *p* ≤ 0.0001 (highly significant). OB—Obino l’Ewai; OR—Orishele; AG—Agbagba; GM—Gonja Manjaya; CAV—Cavendish Williams; DW—Dry weight of sample.

Total tannin concentrations varied significantly (p ≤ 0.0001) across the cultivars and explants. Generally, tannin levels were highest in the root explant and lowest in the pseudostem for all the cultivars tested. The highest levels of tannin were found in roots ranging from 430 ± 13 mg/100 g dry weight in Orishele to 830 ± 12 mg/100 g dry weight in Gonja Manjaya, followed by leaf samples with 270 ± 8 mg/100 g dry weight in Gonja Manjaya to 407 ± 14 mg/100 g dry weight in Obino l’Ewai. However, the lowest concentration of tannin was in pseudostem with a level of 105 ± 9 mg/100 g dry weight in Gonja Manjaya to 318 ± 28 mg/100 g dry weight in Cavendish Williams (Figure 4). 

The concentration of phytochemicals in explants of the same plants was not uniform. The root explant of Gonja Manjaya, Obino l’Ewai, and Cavendish Williams contained enormous amounts of phytochemicals while Orishele and Agbagba had higher antioxidant activity in the pseudostem explant. The varying antioxidant potential to reduce DPPH, a stable free radical, to DPPH-H, a non-radical form in different explants tested revealed the diversity among the cultivars, area of localization, and their potential in combating the effect of oxidative stress due to increase level of reactive oxygen species (ROS). This is made possible because of the innate ability of plant cell machinery to balance the stoichiometry of free radical flux, which is produced when plants are stressed. Chutipaijit [46], showed the correlation between drought tolerance and antioxidant property in rice. The augmentation of this mechanism in the presence of increased oxidative stress result in the production of phenolics, flavonoids, and tannins to confer antioxidant properties [47]. The ability of natural antioxidant agents to scavenge for free radicals that are generated through stresses imposed on plant tissues and eventual tissue injury is essential for in vitro and in situ activities of plants.

The optimum concentration of certain phytochemicals could influence plant responses in vitro. The level of total antioxidants, phenolics and flavonoid content in in vitro plantlets was higher in Cavendish Williams, Gonja Manjaya, and Agbagba compared with Obino l’Ewai and Orishele. There was a probable trend observed in the antioxidant capacity, phenolic content, multiplication rate and the potentiality to produce friable embryogenic calli (FEC) in Agbagba which was higher compared to Obino l’Ewai and Orishele. Likely, the stoichiometry of phenolic quantity and antioxidant potential during in vitro procedures could have supported the production of FEC and multiplication capacity of Agbagba. However, this could be further investigated.

### 2.3. Induction of Embryogenic Callus from Scalps of Plantain 

**Figure 5:**
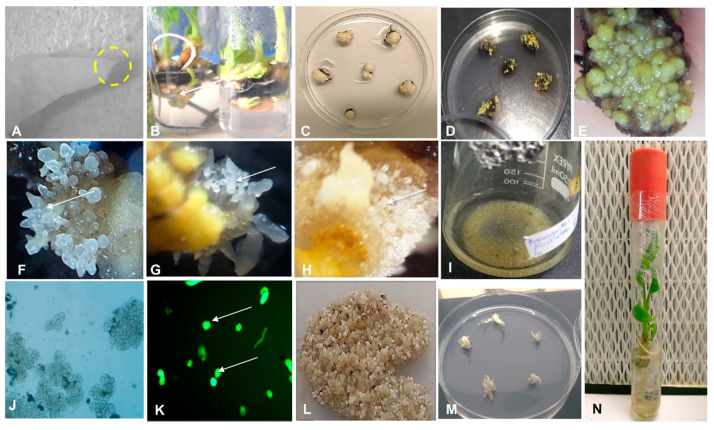
Establishment of embryogenic cell suspension and regeneration of complete plantlet of plantain cultivars Agbagba, Obino l’Ewai, and Orishele. (**A**) Apical meristem (yellow circle showing the portion of shoot tip having apical meristem under 10X magnification); (**B**) Axillary buds (arrow indicating axillary buds); (**C**) Scalps with tiny multiple meristems; (**D**) Callus induced on the scalp; (**E**) Yellowish non-embryogenic callus; (**F**—**H**) Friable embryogenic callus of Orishele, Obino l’Ewai and Agbagba, respectively (arrows showing the embryos under 10X magnification); (**I**) Embryogenic cell suspension; (**J**) Magnified view of cell suspension (40X magnification); (**K**) Fluorescein diacetate stained viable embryogenic cells (1000X magnification); (**L**) Mature embryos; (**M**) Germinating embryos; (**N**) Complete plantlet.

The thin slices of scalps with tiny multiple meristems, developed from axillary buds, cultured on callus induction medium (CIM) in the dark showed initiation of callus on the surface of the explant between 3–4 weeks (Figure 5). The induced calli were either yellowish non-embryogenic or embryogenic at 4–12 weeks. A number of the explants did not respond and turned black as dead scalps (DS). 

No morphological differences were observed in the calli developed for different cultivars of plantain. Several of the scalps produced compact or nodular yellowish non-embryogenic calli (NEC) (Figure 5). Some of these NEC were mucilaginous. The non-embryogenic calli are not suitable for the induction of cell suspension. The scalps cultured on callus induction medium also developed embryogenic calli with individual embryos or FEC with a cluster of many translucent globular and torpedo-like embryos (Figure 5). The FECs are typical callus for developing cell suspension and transferred to the liquid culture medium to generate cell suspension. Although the FECs were generated for all the three cultivars tested, Agbagba produced the highest number of FEC.

The response of the scalps to embryogenic development varied significantly (*p* < 0.0001) across the three plantain cultivars tested, and also depends on the treatment used with various subculture number of the scalp and different media (Table 2). A comparison between the scalp subculture (S) number showed a significant difference (*p* < 0.0001) between S3 and S4, S4 and S5, as well as in S4 and S6 for FEC development. The cultivar influence varied significantly between Agbagba and Obino l’Ewai (*p* < 0.05), Agbagba and Orishele (*p* < 0.0001), and no significant difference observed between Obino l’Ewai and Orishele concerning to their response to generate FEC. Although all the treatments used had the potential of inducing FEC, a significant difference was observed between media ZZ1 and ZZ2 (*p* = 0.007), ZZ1 and ZZ3 (*p* < 0.0001) and no significant difference was observed between ZZ2 and ZZ3. 

For Agbagba, an optimum treatment using ZZ3 (Gamborg B5 medium) supplemented with 1 mg/L 2,4-D (2,4-Dichlorophenoxyacetic acid) resulted in the production of higher percentage of FEC (22 ± 24%) as compared to MS full strength (ZZ1) and MS half-strength (ZZ2) culture media (Table 2). While for Obino l’Ewai and Orishele, the highest FEC percentage was observed in half-strength MS (ZZ2) and full-strength MS (ZZ1), respectively. Regardless of the treatment and subculture cycle employed, FEC was generated at a varying level, and this result is the first optimal report with increased FEC production in comparison to what was previously published [48]. Certain factors such as media type, concentration of growth hormone, age of explant, and genotype are some reported influences on the development of FEC [49,50]. Therefore, optimization may be necessary to obtain the best response to suit specific cultivar. 

The highest efficiency of FEC production (22 ± 24%) for Agbagba was observed at S4 on ZZ3 medium (Table 2). While for Orishele and Obino l’Ewai, the highest percentage of FEC was 9 ± 16% at S4 on ZZ1, and 13 ± 28% at S9 on ZZ2 medium, respectively (Table 2). 

**Figure 6:**
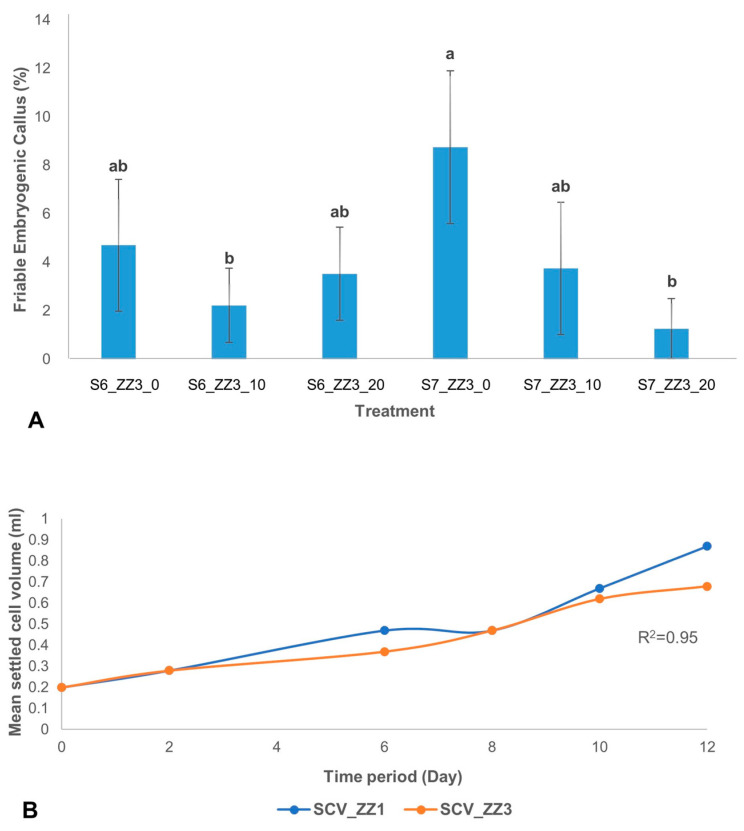
Effect of various media on the development of FEC and proliferation of cell suspension. (**A**) Comparing the effect of various concentration of ascorbic acid on FEC development of Agbagba from scalps at subculture number S6 and S7. The results are presented as percentage mean and standard error of FEC obtained from total number of scalps (*n* ≥ 80) cultured at each subculture number (S6 or S7) in ZZ3 medium (ZZ3_0, ZZ3_10, or ZZ3_20) supplemented with 0, 10, and 20 mg/L ascorbic acid. Different letters (a,b) indicate significant differences in treatment at *p* ≤ 0.05. (**B**) Cell growth of actively proliferating cells of Orishele on different media (ZZ1 and ZZ3) over 12 days. Data is presented as mean of three replicates (*n* ≥ 3).

The effect of ascorbic acid on the development of FEC from scalps showed that at S7, there was a significant difference between treatment with and without ascorbic acid. The highest percentage of FEC development was observed in treatment without ascorbic acid (ZZ3_0) at S7 (Figure 6A). However, at S6 no significant effect of ascorbic acid was observed on FEC development. 

Furthermore, while ascorbic acid is known to improve somatic embryogenesis in some plants [51], it has been observed to hinder the development of somatic embryos in some other plants [52]. In this study, a comparison between scalps of Agbagba at S6 and S7 subjected to culture media containing 0 mg/L, 10 mg/L, and 20 mg/L ascorbic acid indicated a higher production of FEC in medium without ascorbic acid. When in vitro cultures are subjected to stress, the plant initiates its innate ability to balance the ROS produced and quenching capacity through the use of antioxidants. An imbalance of the two components could affect the plant response [53]. Growth hormones like 2, 4-D are known free radical generators used to induce somatic embryogenesis in a plant. In our study, the development of FECs in the absence of ascorbic acid could be that the endogenous antioxidant capacity of Agbagba may be sufficient to quench the ROS generated or the ROS could have signaled the production of FECs. Also, a possible counteractive effect of exogenous antioxidants caused by an imbalance in the reaction of ROS and antioxidant quenching ability could have hindered the production of FEC. A similar result in *Arabidopsis* confirmed that deficiency in ascorbic acid improves somatic embryo development [51]. This is an indication that the absence of ascorbic acid does not have any negative influence on FEC development in Agbagba. However, this could be further investigated.

The influence of the phenolic compound on induction of SE is cultivar dependent. Phenol rich compounds have been shown to improve the induction of SE in *Feijoa sellowiana* [29], whereas, it inhibits SE in *Coffea canephora* [13]. The high level of phenolics in Agbagba as compared to Obino l’Ewai and Orishele may contribute towards its improved response to developing FECs. On the other hand, the response of Obino l’Ewai to SE is relatively higher than Orishele. This difference may be attributed to the total phenolic content, which is higher in Obino l’Ewai compared to Orishele. The genotypic dependency of SE suggests an evaluation of phytochemicals in genotypes before the optimization of SE.

### 2.4. Generation of Embryogenic Cell Suspension (ECS) 

FECs of Agbagba obtained from ZZ1, ZZ2, and ZZ3 treatments were subjected to the same respective liquid media type (i.e., ZZ1, ZZ2, ZZ3) to determine optimum culture medium for initiation of cell suspension. One cluster of FEC was transferred to a conical flask having 5 mL of liquid medium. The effect of reduced concentration of 2, 4-D (0.5 mg/L) on generation of embryogenic cells was also observed for the three treatment types. Cultures were refreshed every 10 days for 3 months when a full proliferation of cells was attained. At three months after initiation, the settled cell volume of cell suspension was measured. The optimum cell volume (3.3 mL) was observed in ZZ2 with 0.5 mg/L 2, 4-D (ZZ2 ½). Although ZZ3 (B5 media type) seems to enhance cell multiplication to a certain extent, however, cells on this medium became nodular and not fit for subsequent experiments. ZZ1 and ZZ2 (MS media type) either with 1 mg/L or 0.5 mg/L 2, 4-D supported the proliferation of ECS of Agbagba in the liquid medium.

The viability of embryogenic cells of plantain was estimated by staining with fluorescein diacetate (FDA) dye, which fluoresces green light when absorbed by live cells using a flow cytometer. The viability of embryogenic cells was different in both types of media. At the start of the culture (day 0), 35 ± 0.9% cells were observed to be viable on B5 medium compared to 12 ± 3% viable cells on MS medium. At day 4 post-culture initiation, the higher value of viable cells (71 ± 2%) was observed on B5 medium compared to 27 ± 7% viable cells on MS medium. However, at day 8 post-culture initiation, the viability of cells (62 ± 0.7%) decreased at B5 medium, whereas the viability of cells (28 ± 14%) increased on MS medium. 

Further, the proliferation of ECS was measured. An aliquot of 0.2 mL settled cell volume (SCV) of the actively proliferating cell was further used to monitor cell growth over 12 days by determining its settled cell volume. Cell proliferation was monitored for 12 days in cells cultured in MS (ZZ1) and B5 (ZZ3) media. In both culture media, although there was a correlation (R^2^ = 0.95) between the two types of culture media used for cell growth as measured by the SCV, MS medium (ZZ1) produced finer cells. In contrast, nodular cells were observed in B5 medium (ZZ3) (Figure 6B). 

A complete regeneration to a whole plant was achieved from the ECS of all plantain cultivars tested (Figure 5). Regeneration capacity of ECS was determined by counting the plantlets generated from embryos of plantain cells. A total of 7642, 11,269 and 4082 plantlets were obtained from 0.5 mL SCV for Agbagba, Obino l’Ewai, and Orishele, respectively.

### 2.5. Agrobacterium-Mediated Transformation and Regeneration of Transgenic Events of Plantain 

**Figure 7:**
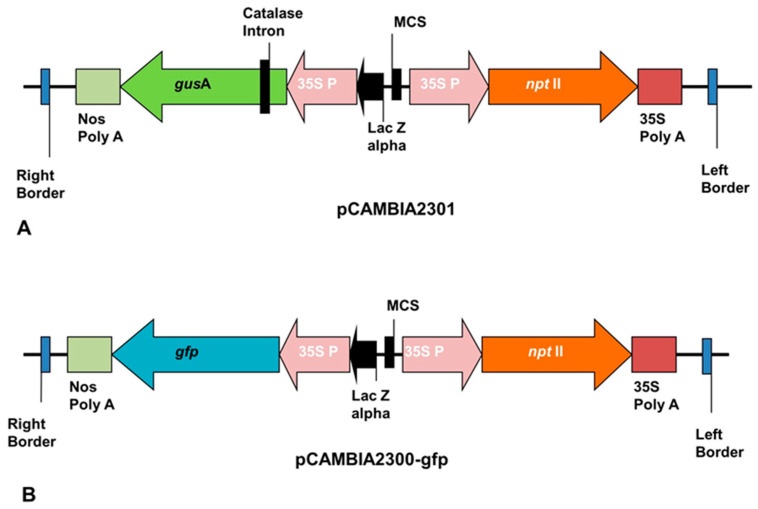
Map of plasmid construct. (**A**) pCAMBIA2301; (**B**) pCAMBIA2300-*gfp.*

The ECS of plantain generated were successfully transfected with *Agrobacterium tumefaciens* strain EHA105 harboring pCAMBIA2301 or pCAMBIA2300-*gfp* containing *npt*II as selection marker and *gusA* or *gfp* gene as a reporter gene (Figure 7). 

**Figure 8:**
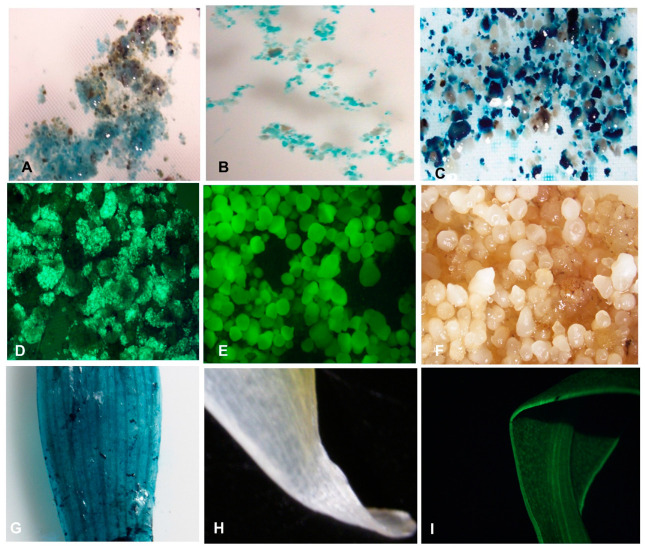
Transient and stable expressions of *gusA* and *gfp* in transformed plantain. (**A**–**C**) Transient expression of *gusA* gene in *Agro*-infected embryogenic cell suspension (ECS) of different cultivars, (**A**) Obino l’Ewai; (**B**) Orishele; (**C**) Agbagba; (**D**) Transient expression of *gfp* gene in the *Agro*-infected ECS of Obino l’Ewai; (**E**) Expression of *gfp* gene in mature embryos of Obino l’Ewai; (**F**) Control non-transformed embryos of Obino l’Ewai; (**G**) Stable expression of *gusA* gene in leaf explant of the transgenic plant of Obino l’Ewai; (**H**) Control leaf from non-transgenic plant of Obino l’Ewai; (**I**) Stable expression of *gfp* gene in leaf explant of the transgenic plant of Obino l’Ewai. Photographs (**A**–**I**) were taken using stereomicroscope (SMZ1500) with 10X magnification.

The transformation capacity of cell suspension generated for all three cultivars was tested through transient expression of *gus*A reporter genes in the *Agro*-infected cells after 2-days or 3-days of co-cultivation. The blue coloration as the transient expression of *gus*A gene confirmed the transformability of ECS of the three plantain cultivars through *Agrobacterium*-mediated transformation (Figure 8A–C). The blue pigmentation observed when X-gluc reacted with beta-glucuronidase (GUS), an enzyme produced by the *gus*A gene in the transformed ECS, is an indication of expression of the reporter gene in the plant cells. The higher expression of the *gus*A gene was observed in cells with treatment T2-T5 (with acetosyringone) compared to T1 (no acetosyringone) at both 2-days and 3-days co-cultivation of ECS with *Agrobacterium* (Supplementary Figure S1). This result suggested that the use of acetosyringone during the transformation process is required for efficient transformation. 

**Figure 9:**
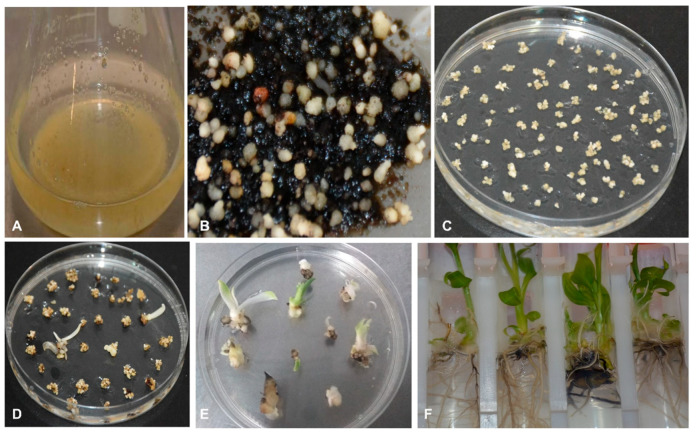
Transformation and regeneration of putative transgenic events of plantain cultivar Obino l’Ewai. (**A**) Cell suspension ready to be transformed; (**B**) *Agro*-infected cells showing the development of embryos on selective medium containing kanamycin; (**C**) Embryo maturation on selective medium; (**D**,**E**) Embryos germinating on selective media; (**F**) Fully regenerated putative transgenic events.

*Agro*-infected cells of Obino l’Ewai were further progressed for generation of stably transformed plants. The *Agro*-infected cells developed embryos on embryo development medium (EDM) supplemented with kanamycin while non-transformed cells turned black. Selected transformed embryos further germinated on proliferation medium and individual plantlets developed into complete rooted plantlets in the selective medium containing kanamycin after one month in culture (Figure 9). 

A total of 141 fully developed kanamycin-resistant putative transgenic events of Obino l’Ewai were obtained in a single transformation experiment across all the treatments. The transformation efficiency was calculated based on the number of PCR positive events per mL SCV. The influence of the co-cultivation period and concentration of acetosyringone showed that a co-cultivation period of 2-days and 3-days was optimum for *Agrobacterium*-mediated transformation of plantain cultivars. Furthermore, low acetosyringone concentration of 50 µM was equally effective in mediating transfection of plantain cells. The maximum transformation efficiency of 38 events per mL SCV was observed for treatment T2 with 2-days co-cultivation with plasmid construct pCAMBIA23000-gfp (Table 3). For *gfp*-T4-2-day co-cultivation period, 15 PCR positive transgenic events were obtained from 0.5 mL SCV. The transformation with pCAMBIA2301 with *gus*A reporter gene showed maximum efficiency for treatment T4. The number of transgenic events generated for *gusA* at 2-days and 3-days co-cultivation period for treatment T4 was 15 and 9 PCR positive events from 0.5 mL SCV, respectively (Table 3). 

The regenerated transgenic events were validated by expression of reporter genes using histochemical GUS assay, UV-stereomicroscopy for GFP, and molecular characterization to confirm the presence and integration of the transgene.

### 2.6. Expression of Reporter Genes

The transient and stable expressions of the *gfp* and *gusA* reporter gene confirmed that ECS of all the three plantain cultivars were transformable. The images of the gene expression of *gfp* and *gusA* were micrographed using a stereomicroscope (Figure 8).

### 2.7. Molecular Characterization of Transgenic Events

#### 2.7.1. Genomic DNA Isolation and PCR Analysis

**Figure 10:**
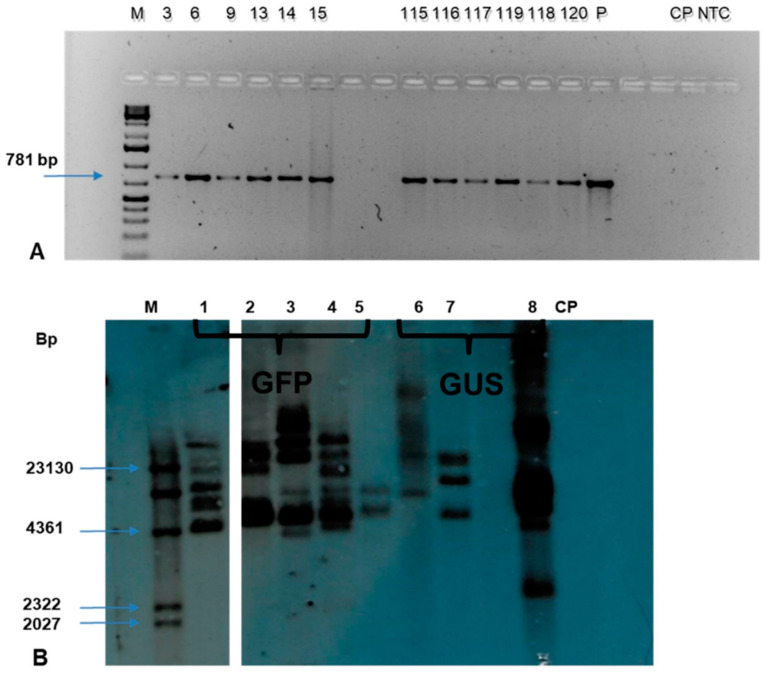
Molecular analysis of transgenic events of plantain cultivar Obino l’Ewai. (**A**) PCR analysis using *npt*II specific primers. **M**—DNA ladder sample; **3**–**15**—*gfp* transgenic events; **115**–**120**—*gusA* transgenic events; **P**—plasmid DNA as a positive control; **CP**—Non-transgenic control plant; **NTC**—non-template control; (**B**) Southern blot analysis of selected transgenic events using *npt*II specific probe. M: DIG molecular marker; Samples (1–8)—Transgenic events (GFP event T2-2D, GFP event T4-2D, GFP event T4-3D, GFP event T5-2D, GFP event T5-3D, GUS event T2-3D, GUS event T4-2D, GUS event T4-3D); CP- Non-transgenic control plant.

Genomic DNA was isolated from 141 putative transgenic events using the Cetyltrimethyl ammonium Bromide (CTAB) method. The quantification was done using a Nanodrop spectrophotometer while the quality was determined on 1% agarose gel electrophoresis. The PCR analysis of genomic DNA using *npt*II specific primers confirmed the presence of the transgene in 115 transgenic events. The expected amplicon size of 781 bp was obtained in all the PCR positive events confirming the presence of transgenes in the genome of individual transgenic plants tested. (Figure 10A). In contrast, no amplicon was observed in non-transgenic control plant. 

#### 2.7.2. Southern Blot Analysis

The Southern blot analysis of the selected events across the treatment confirmed the integration of transgene in the transgenic events tested (Figure 10B). The *Hin*dIII restriction enzyme has a single restriction site in the pCAMBIA2301 and pCAMBIA2300-*gfp* constructs. The eight transgenic events tested showed positive bands with a copy number of 2-7; however, no band was observed in the non-transgenic control plant.

Confirming the transformability of the embryogenic plantain cells is essential before downstream experimental procedures for crop improvement. In this study, the transformation ability of the embryogenic cells generated for all the three plantain cultivars was determined through *Agrobacterium*-mediated transformation using the *gusA* reporter gene. The transient expression of the reporter genes indicated the transformability of the plantain cells generated. The transformation capability was further confirmed with the stable transformation of the cells of plantain cultivar Obino l’Ewai using *gfp* and *gusA* genes and molecular characterization of the transgenic events. 

The influence of acetosyringone, an analogue to the type of phenolic compound secreted when dicot plants are wounded, was observed on the transformation efficiency of the ECS of plantain. This compound is known to activate the virulence (*vir*) gene in *Agrobacterium* by binding to the receptor of the cell, hence enabling the transfer of the T-DNA into the plant cell [54]. The synthetic form of this naturally occurring compound is employed during *Agrobacterium*-mediated transformation of dicots like cotton [55] and monocot plants like rice [56], wheat [57] and *Musa* spp. [41,42,58] to improve its transformation efficiency. About 100-250 µM acetosyringone has been successfully reported for bananas [37,38,41,42,58]. Although, this work has shown that a lower concentration of 50 µM acetosyringone in co-cultivation medium can be used to achieve transformation in Obino l’Ewai. A higher level of 200 µM and 400 µM acetosyringone is equally efficient in transforming plantain cells within 2-days of the co-cultivation period, which is similar to the previous reports [41,42,58]. 

The co-cultivation period also influences the efficiency of *Agrobacterium*-mediated transformation of plantain cells. Although the 2-days co-cultivation period favoured the generation of more transformed plants, the 3-days co-cultivation period was also beneficial and doesn’t impose any negative influence on transformation efficiency. This similar observation was also confirmed as an optimum co-cultivation of 2-days in the transformation of tobacco [59] and cucumber [60]. However, both authors did not state if acetosyringone was used to improve the transformation efficiency. Also, previous work on banana suggested an optimum co-cultivation period of 3-days and 4-days [41,42,61,62].

Transient expression analysis by GUS assay showed that acetosyringone is essential for the successful transfection of plantain cells. Varying intensity of blue colouration as marked by expression of *gusA* gene showed the influence of acetosyringone and co-cultivation period of *Agrobacterium* with plantain cells studied. As depicted by the transient expression of the reporter genes, a transfection period of 3-days may allow more cells to be transformed.

Southern blot analysis confirmed the integration of both transgenes in the plant as well as the independence of the events generated, as established by the varying copy numbers. The result also confirmed that the successful transformation of plantain cells could be obtained either at 2-days or 3-days of co-cultivation period and low concentration (50 µM) of acetosyringone in the co-cultivation medium for plantain transformation.

## 3. Materials and Methods 

### 3.1. Plant Material and Preparation

Three cultivars (Agbagba, Orishele and Obino l’Ewai) of plantain (AAB genome) were obtained from the field gene bank of the International Institute of Tropical Agriculture (IITA), Nigeria and established in IITA Nairobi. Plantain cultivar Gonja Manjaya (AAB) and dessert banana (AAA genome) cultivar Cavendish Williams used in this study were obtained from the Plant Transformation laboratory and field collection of IITA, Nairobi, Kenya. 

Meristem cultures of plantain (*n* ≥ 3) were initiated from suckers as described by Gueye et al., [63]. Samples were screened for the absence of any virus and bacteria following the procedure described by Strosse et al., [64]. Clean pathogen-free plantlets were multiplied, and multiple buds were induced as described by Tripathi et al. [41]. 

### 3.2. Quantification of Targeted Phytochemicals in Banana and Plantain Cultivars

Composite samples were obtained from 15 representatives of in vitro plants per cultivar obtained from the ascorbic acid-free culture media and field explants (root, young leaf and pseudostem) from 3 representatives of field-grown plants of banana and plantain cultivars. The samples were collected separately in a perforated, labelled sample bags, kept overnight at −80 °C and freeze-dried (Christ Alpha 2-4LSCplus, Osterode am Harz, Germany) for 72 h. Dried samples were pulverized to a fine powder with a grinder (Waring commercial blender 8010ES, model HGBTWTS3, Torrington, CT, USA) in a fume hood. Three replicate samples per explant were subjected to the extraction process depending on the phytochemical to be determined. 

#### 3.2.1. Determination of Antioxidant Content, Total Phenolics, and Flavonoids 

Sample extraction was done by addition of 10 mL of 80% methanol to 50 mg pulverized sample in a 50 mL falcon tube and placed on a mechanical shaker (New Brunswick Scientific, Edison, NJ, USA) for 24 h at 25 °C. The sample mixture was then centrifuged at 4000 rpm for 10 min and the supernatant aliquots were taken for determination of total flavonoid, phenol and antioxidant content in plant samples. Antioxidant capacity was determined by assaying the scavenging activity of 2, 2-diphenyl-1-picrylhydrazyl (DPPH), a stable free radical to give rise to a non-radical form DPPH-H (Diphenylpicrylhydrazine) in a photometric analysis. The reaction mixture was prepared by pipetting components as stated in Supplementary Table S1 in a microtiter plate, incubated for 30 min at room temperature and absorbance read at 515 nm in a spectrophotometer plate reader (Biotek Synergy HT Gen5 1.11, Winooski, VT, USA). The total antioxidant capacity as determined by DPPH scavenging affinity was calculated from the calibration curve (R^2^ = 0.98) and expressed as mg of Trolox equivalent (TE) equivalent per 100 g of the sample collected from the field and in vitro plantlets.

Total phenol content was determined using a modified Folin–Ciocalteu protocol [65]. The modification was in the reduction of reaction volume to fit the microtiter plate reader. Reagents were added sequentially (Supplementary Table S2) and mixed by priming. The reaction was covered with aluminum foil and allowed to incubate for 90 min at room temperature. Absorbance was then read at 725 nm in a microtiter plate spectrophotometer reader (Biotek Synergy HT Gen5 1.11, Winooski, VT, USA). The total phenolic content was calculated from the calibration curve (R^2^ = 0.98) as the gallic acid equivalent (GAE) per 100 g sample.

Flavonoid content in explants was determined using aluminum chloride colorimetric method [66]. The reaction mixture was prepared as outlined in Supplementary Table S3, incubate at room temperature for 30 min before reading at 510 nm on a microtiter spectrophotometric plate reader (Biotek Synergy HT Gen5 1.11, Winooski, VT, USA). The total flavonoid content (R^2^ = 0.99) was calculated as a catechin equivalent.

#### 3.2.2. Determination of Total Tannins

The tannin content in banana and plantain cultivars were determined using a modified Folin–Denis protocol [67,68,69]. The modification was in the reaction volume used to fit the microtiter reader. About 50 mg of each sample was weighed in triplicate in sterile 50 mL falcon tubes and labelled appropriately. A volume of 7.5 mL distilled water was added to each sample and heated gently at 85 °C in a water bath for 1 h. The volume of each sample was made up to 10 mL by adding distilled water. An aliquot of 1 mL extract was placed in a clean 1.5 mL Eppendorf tube and centrifuged for 10 min at 14,000 rpm. Using a microtiter plate as outlined in Supplementary Table S4, samples, standards and Folin–Denis solution were pipetted in respective wells and mixed gently by priming. After 5 min, 7% Na_2_CO_3_ was added to the whole mixture and mixed gently by priming. The plate was covered with aluminum foil and the reaction was allowed to incubate at room temperature for 30 min. The absorbance of the reactions was read at 700 nm in a microtiter plate spectrophotometer reader (Biotek Synergy HT Gen5 1.11, Winooski, VT, USA). The total tannins as extrapolated from the calibration curve (R^2^ = 0.99) was determined as a tannic acid equivalent.

### 3.3. Development of Embryogenic Cell Suspension

A conglomerate of tiny meristem called scalps, subsequent callus induction and cell suspension initiation, were done using the modified protocol of Strosse et al., [64] and Tripathi et al. [41]. The modification involved the use of full-strength MS-based medium ZZ1 (MS salt and vitamins, 10 mg/L ascorbic acid, 1 mg/L 2,4-D, 219 µg/L zeatin, 30 g/L sucrose, 3 g/L gelrite, pH: 5.8), half-strength MS-based medium ZZ2 (½ MS salts and vitamins, 10 mg/L ascorbic acid, 1 mg/L 2,4-D, 219 µg/L zeatin, 30 g/L sucrose, 3 g/L gelrite, pH: 5.8) and B5 based medium ZZ3 (Gamborg B5 salt and vitamins, 10 mg/L ascorbic acid, 1mg/L 2,4-D, 219 µg/L zeatin, 30 g/L sucrose, 3 g/L gelrite, pH: 5.8). The cultures were kept in the dark at 26 ± 2 °C. Generation of embryogenic cell suspensions was optimized, followed by routine experiments of which a total of 1919, 749, and 1220 scalps were used from plantain cultivars Agbagba, Obino l’Ewai and Orishele, respectively. The number of friable embryogenic calli (FEC), the yellowish non-embryogenic calli, and dead scalps explant were recorded. Embryogenic cell suspensions (ECS) were initiated from FEC of all the three cultivars and maintained for about 10–12 months in liquid culture. The initiation of ECS in liquid culture was done routinely to ensure the availability of fresh ECS.

The effect of ascorbic acid on development of FEC from scalps of plantain was tested by culturing the scalp explants of Agbagba at subculture number 6 and 7 (S6 and S7) on ZZ3 medium supplemented with 0, 10, and 20 mg/L ascorbic acid at 26 ± 2 °C in dark. A total of 80 scalps were used for each treatment at S7, while at S6, a total of 85, 90 and 85 scalps were subjected to 0, 10, and 20 mg/L ascorbic acid, respectively. 

### 3.4. Testing of the Viability of ECS by Cell Proliferation Rate and FDA Staining

The cells were stained with fluorescein diacetate (FDA), and the density of live stained cells was measured using a flow cytometer. Cells were sieved using a 40-micron, and 1 mL of the homogenous cells were aliquoted in a 1.5 mL tube and centrifuged at 1500 rpm for 5 min. The supernatant was discarded, and the pellet re-suspended in 100 µL phosphate-buffered saline (PBS) solution. An aliquot of 30 µL was dispensed in a new 1.5 mL Eppendorf tube, 2 µL of staining solution (FDA) was added to the cells, mixed by pipetting and allowed to incubate at room temperature for 15 min. This was followed by washing with PBS buffer, resuspension in 200 µL PBS, and acquired using flow cytometry to measure the number of cells that fluoresced after staining with the FDA dye. 

The ECS proliferation rate was measured using 0.2 mL SCV as a starting material for 12 days at intervals of 2-days. The experiments were done in triplicate. 

### 3.5. Regeneration of Complete Plantlets from Embryogenic Cell Suspension

The regeneration capacity of ECS was determined as described by Tripathi et al., [41] using 0.5 mL settled cell volume (SCV). 

Cell suspensions for each cultivar were sieved through a 1000-micron sterile metal sieve. About 0.5 mL SCV of each cell event of Agbagba, Orishele, and Obino l’Ewai were measured in sterile 15 mL falcon tube and diluted with ZZ medium to 10 mL volume and transferred onto sterile mesh placed on sterile paper towels to absorb the liquid media. The mesh containing the plant cells were placed on embryo development media (EDM: 3.2 g/L Schenk & Hidebrandt (SH) salts, 1X MS vitamins, 60 mg/L ascorbic acid, 0.1 mg/L zeatin, 100 mg/L glutamine, 100 mg/L malt extract, 1 mg/L biotin, 230 mg/L proline, 60 mg/L citric acid, 400 mg/L L-cysteine, 0.2 mg/L NAA, 0.2 mg/L 2ip, 0.2 mg/L kinetin, 10 g/L lactose, 45 g/L sucrose, 3 g/L gelrite, pH: 5.8) in petri dishes and refreshed every 2 weeks for 8 weeks. Developed embryos were regenerated to whole plantlets as described by Tripathi et al. [41]. The embryo regeneration capacity was determined by the number of plantlets per mL of SCV.

### 3.6. Agrobacterium-Mediated Transformation of Plantain Using ECS

The transformation procedure, which included preparation of bacterial culture, preparation of explant for transformation, and co-cultivation was done using a modified protocol as described by Tripathi et al. [41] and regeneration as described by Tripathi et al. [42]. The modifications were in the bacterial re-suspension medium (BRM: 1/10X MS macro, 1X MS micro (100x), 1X MS vitamins, 9 mg/L thiamine, 400 mg/L L-cysteine, 36 g/L glucose, 68.5 g/L sucrose, acetosyringone (0, 50, 100, 200, 400 µM) pH: 5.3) and bacteria co-cultivation medium (BCCM: 1/10X MS Macro, 1X MS Micro, 2X MS vitamins + myoinositol, 30 g/L maltose, 400 mg/L cysteine, 300 mg/L proline, 100 mg/L glutamine, 100 mg/L malt extract, 1 mg/L biotin, 10 mg/L ascorbic acid, 30 g/L sucrose, 10g/L glucose, acetosyringone (0, 50, 100, 200, 400 µM), 10 g/L gelrite, pH: 5.5). The *Agrobacterium tumefaciens* culture harboring plasmid construct was pre-induced for 2–3 h in BRM containing different concentrations of acetosyringone. After 3 h, the optical density (OD_600 nm_) of *Agrobacterium* culture was adjusted to 0.5 and used for infecting the ECS for 60 min at 30 rpm on a rotary shaker. The *Agro*-infected ECS were then co-cultivated on BCCM with varied concentrations of acetosyringone for 2-days and 3-days. *Agrobacterium*-mediated transformation employed the use of *Agrobacterium* strain EHA 105 harboring pCAMBIA2300-*gfp* and pCAMBIA2301 containing *neomycin phosphotransferase* II (*nptII*) gene as selection marker and *gfp* or *gusA* as a reporter gene (Figure 7). After co-cultivation, the transient expression of *Agro*-infected ECS was checked for all three cultivars Agbagba, Orishele, and Obino l’Ewai. The *Agro*-infected ECS of cultivar Obino l’Ewai were regenerated on selective medium containing kanamycin to generate transgenic events following the procedure of Tripathi et al. [42]. Although stable transformation experiment with the reporter genes was done once, more than three experiments were performed for transient expressions of reporter genes to confirm the transformability of the plantain cells. 

### 3.7. Histochemical GUS Assay and Transient Expression of the gfp Gene

Histochemical GUS assay of *Agro*-infected ECS and leaf explant of transgenic plants was carried out as described by Jefferson et al. [70] and Tripathi et al. [41]. Transformed explants expressing *gus*A gene were micrographed using a stereomicroscope (SMZ1500, Tokyo, Japan), and *gfp* expressions were viewed under UV stereo microscope (SMZ1500, Tokyo, Japan).

### 3.8. Molecular Characterization of Transformed Plants

#### 3.8.1. Genomic DNA Extraction

Genomic DNA was extracted from putative transgenic events plant using the modified CTAB method as described by Tripathi et al. [42]. Quantity and quality of DNA were determined using a Nanodrop (ThermoScientific Nanodrop 2000 spectrophotometer, Wilmington, NC, USA) and 1% (w/v) agarose gel electrophoresis respectively.

#### 3.8.2. PCR Analysis

The putative transgenic events were analyzed for the presence of the transgene by PCR analysis using primers specific to the *npt*II gene (*npt*II_F: GATGGATTGCACGCAGGTTCTC, *npt*II_R: AGAAGAACTCGTCAAGAAGGC). A positive (plasmid DNA) and non-transgenic control plant and plasmid DNA were included as controls in the experiment. A 20 µL reaction mix containing (Hot Star Taq master mix: 10 µL, *npt*II F and R primer (10 µM): 1 µL each, Nuclease free water: 6 µL, DNA template: 2 µL (25 ng)) was prepared and incubated in a thermocycler using the following PCR conditions to amplify the primer specific sites (95 °C: 15 min, 94 °C: 30 s, 68.2 °C: 30 s, 72 °C: 1 min (25 cycles), 72 °C: 5 min, 4 °C: ∞). The amplified PCR products were resolved by electrophoresis on a 0.8% (w/v) agarose gel.

#### 3.8.3. Southern Blot Analysis

PCR positive events were selected randomly for Southern blot analysis to determine the copy numbers of the transgene integrated into the plant genome. The procedure was done as described by Tripathi et al. [42], a restriction digest using 20 µg of genomic DNA restricted with 5 µL *Hin*dIII enzyme HF was done. Fragments were resolved by electrophoresis on a 0.8% (w/v) agarose gel and transferred onto a positively charged nylon membrane. The membrane was hybridized with DIG-labelled probe specific to *npt*II and detected by CDP star, a chemiluminescent substrate.

### 3.9. Statistical Analysis

The Statistical analysis was done using the generalized linear model (PROC GLM and PROC GENMOD) was analyzed using the Statistical Analysis System (SAS V9.4, Cary, NC, USA) to obtain the variance components, multiple comparison and analysis of likelihood parameter estimates. The least significant means (LSMEANS and least significance difference (LSD) was used to compare the means across different cultivars. The Flowjo version 5.4 (Ashland, OR, USA) was used to analyze the cell count from the flow cytometer.

## 4. Conclusions

Genetic transformation is a valuable tool for crop improvement particularly for the traits which are not available in the germplasm. Here, we report *Agrobacterium*-mediated transformation and regeneration of economically significant cultivars of plantain, which are commonly grown by smallholder farmers in West Africa. Quantification of phytochemicals found in plantain could be useful for optimizing somatic embryogenesis and production of FEC that is preferred explants for transformation. This study reports the development of embryogenic cell suspensions (ECS) and *Agrobacterium*-mediated transformation from three farmer-preferred plantain cultivars, Agbagba, Obino l’Ewai, and Orishele. 

## Figures and Tables

**Table 1:** The proliferation potential of various cultivars of plantain at different sub-culturing on proliferation medium.

Cultivar	Number of Shoots Per Explant at Various Sub-culturing Stage		
	**S1**	**S2**	**S3**
**Agbagba**	1.6 ± 0.5 ^ns^	29.4 ± 8.2 ***	119.0 ± 42.2 ***
**Obino l’Ewai**	2.0 ± 1.1 ^ns^	20.4 ± 11.5 ***	49.4 ± 25.9 ***
**Orishele**	2.5 ± 0.7 ^ns^	25.0 ± 1.4 ***	79.5 ± 7.8 ***

**Note.** Data are presented as mean and standard deviation of number of shoots per explant. ^ns^ indicates non-significant (*p* > 0.05) difference in number of shoots per explant among different cultivars at subculture S1. *** indicates highly significant (*p* ≤ 0.001) difference in number of shoots per explant among different cultivars at subculture S2 and S3.

**Table 2:** Efficiency of embryogenic callus development from scalps of West African plantain cultivars, Agbagba, Obino l’Ewai, and Orishele, on different treatments.

Cultivar	Treatment	Friable Embryogenic Calli (FEC) (%)	Non-Embryogenic Calli (NEC) (%)	Dead Scalp (DS) (%)
**Agbagba**	S2_ZZ1	9 ± 11^bc^	20 ± 16^g^	71 ± 20^ab^
	S2_ZZ2	0 ± 0^e^	97 ± 8^a^	3 ± 8^g^
	S2_ZZ3	15 ± 21^ab^	25 ± 18^g^	60 ± 26^bc^
	S3_ZZ1	2 ± 6^cde^	50 ± 30^ef^	48 ± 29^c^
	S3_ZZ2	5 ± 13^cde^	90 ± 21^ab^	5 ± 11^g^
	S3_ZZ3	8 ± 11^bcd^	67 ± 22^cde^	26 ± 23^def^
	S4_ZZ1	8 ± 15^bcde^	49 ± 30^ef^	42 ± 32^cde^
	S4_ZZ2	15 ± 20^a^	81 ± 20^bc^	4 ± 8^g^
	S4_ZZ3	22 ± 24^a^	53 ± 33^ef^	25 ± 24^ef^
	S5_ZZ1	0 ± 0^e^	26 ± 31^g^	74 ± 31^a^
	S5_ZZ2	7 ± 11^bcde^	72 ± 26^cd^	22 ± 27^f^
	S5_ZZ3	5 ± 9^cde^	64 ± 30^cde^	31 ± 32^def^
	S6_ZZ1	8 ± 14^bcde^	37 ± 20^fg^	56 ± 22^bc^
	S6_ZZ2	2 ± 7^cde^	80 ± 21^bc^	18 ± 21^fg^
	S6_ZZ3	1 ± 5^de^	55 ± 38^def^	44 ± 37^cd^
***p*-value**		***	***	***
**Obino l’Ewai**	S3_ZZ1	0 ± 0^b^	89 ± 10^ab^	11 ± 10^de^
	S3_ZZ2	5 ± 9^ab^	95 ± 9^a^	0 ± 0^e^
	S3_ZZ3	8 ± 15^ab^	77 ± 29^abcd^	15 ± 29^cd^
	S4_ZZ1	0 ± 0^b^	60 ± 25^bcde^	40 ± 25^ab^
	S4_ZZ2	0 ± 0^b^	96 ± 9^a^	4 ± 9^de^
	S4_ZZ3	4 ± 9^ab^	56 ± 17^efg^	40 ± 14^ab^
	S5_ZZ1	0 ± 0^b^	60 ± 19^defg^	40 ± 19^ab^
	S5_ZZ2	1 ± 5^b^	87 ± 20^ab^	12 ± 20^de^
	S5_ZZ3	8 ± 21^ab^	64 ± 30^bcdef^	27 ± 25^bc^
	S6_ZZ1	0 ± 0^b^	43 ± 8^fg^	57 ± 8^a^
	S6_ZZ2	10 ± 15^ab^	90 ± 15^ab^	0 ± 0^e^
	S6_ZZ3	0 ± 0^b^	97 ± 8^a^	3 ± 8^de^
	S8_ZZ1	5 ± 10^ab^	40 ± 33^g^	55 ± 34^a^
	S8_ZZ2	5 ± 10^ab^	85 ± 19^abc^	10 ± 12^cde^
	S8_ZZ3	0 ± 0^b^	90 ± 12^ab^	10 ± 12^cde^
	S9_ZZ1	0 ± 0^b^	92 ± 11^ab^	8 ± 11^de^
	S9_ZZ2	13 ± 28^a^	80 ± 28^abcd^	8 ± 15^de^
	S9_ZZ3	0 ± 0^b^	87 ± 12^abcd^	13 ± 12^cde^
***p*-value**		ns	***	***
**Orishele**	S2_ZZ1	0 ± 0^c^	57 ± 29^cd^	43 ± 29^bcd^
	S2_ZZ2	7 ± 10^ab^	93 ± 10^a^	0 ± 0^f^
	S2_ZZ3	0 ± 0^c^	60 ± 42^cd^	40 ± 42^bcd^
	S3_ZZ1	0 ± 0^c^	39 ± 28^de^	61 ± 28^ab^
	S3_ZZ2	4 ± 12^abc^	89 ± 19^ab^	7 ± 10^ef^
	S3_ZZ3	4 ± 8^abc^	53 ± 25^dc^	44 ± 27^bcd^
	S4_ZZ1	9 ± 16^a^	49 ± 34^cd^	42 ± 37^bcd^
	S4_ZZ2	2 ± 6^bc^	69 ± 23^bc^	29 ± 24^cde^
	S4_ZZ3	2 ± 6^bc^	57 ± 24^cd^	42 ± 26^bcd^
	S5_ZZ1	1 ± 4^bc^	50 ± 44^cd^	49 ± 44^bc^
	S5_ZZ2	0 ± 0^c^	86 ± 24^ab^	14 ± 24^ef^
	S5_ZZ3	2 ± 6^bc^	58 ± 27^cd^	41 ± 29^bcd^
	S6_ZZ1	0 ± 0^c^	26 ± 27^e^	74 ± 27^a^
	S6_ZZ2	3 ± 7^bc^	70 ± 33^abc^	27 ± 34^de^
	S6_ZZ3	1 ± 5^bc^	59 ± 26^cd^	40 ± 28^bcd^
***p*-value**		**	***	***

**Note:** S: Subculture number, ZZ1: MS-based medium, ZZ2: Half-strength MS-based medium, ZZ3: B5 based medium. Data are presented as percentage mean and standard deviation of friable embryogenic calli (FEC), non-embryogenic calli (NEC) or non-responding explants as dead scalps (DS) initiated from scalps (n = 749, 1220, 1919 for Obino l’Ewai, Orishele and Agbagba, respectively) with different subculture number cultured on various media. Different letters in the same column for each cultivar indicate differences in FEC, NEC, or DS. ns, non-significant (*p* > 0.05), ** very significant (*p* ≤ 0.01), *** highly significant (*p* ≤ 0.0001).

**Table 3:** Transformation efficiency of embryogenic cell suspension (ECS) of plantain cultivar Obino l’Ewai with varying concentration of acetosyringone and co-cultivation period.

Construct	Treatment	Co-Cultivation Period	No. of Plant Generated on Selective Medium	No. of PCR Positive PlantsGenerated from 0.5 mL SCV	Transformation Efficiency Plantlets/mL SCV
**pCAMBIA2300-gfp**	T2	2D	30	19	38
	T2	3D	17	14	28
	T3	2D	13	10	20
	T3	3D	2	1	2
	T4	2D	16	15	30
	T4	3D	15	12	24
	T5	2D	11	10	20
	T5	3D	11	8	16
**pCAMBIA2301**	T2	3D	2	2	4
	T4	2D	15	15	30
	T4	3D	9	9	18

**Note:** T2-T5-Treatment with various concentration (50, 100, 200, 400 µM) of acetosyringone, 2D: 2-days co-cultivation period, 3D: 3-days co-cultivation period. Transformation efficiency = No. of PCR positive transgenic events/mL SCV.

## Supplementary Materials

The following are available online at https://www.mdpi.com/2223-7747/9/6/789/s1, Figure S1: Transient expression of *gusA* gene across the various treatments and co-cultivation periods of *Agrobacterium*-mediated transformation of different plantain cultivars; Agbagba, Obino l’Ewai, and Orishele, Table S1: Various components of reaction for determination of total antioxidants, Table S2: Components of reaction mix for determination of total phenolics, Table S3: Reaction mixture for determination of total flavonoids, Table S4: Reaction mixture for determination of Tannin content.
