# Supplementary material for: Phytochemical Analysis and Establishment of Embryogenic Cell Suspension and Agrobacterium-mediated Transformation for Farmer Preferred Cultivars of West African Plantain (Musa spp.)
Source: Plants (Basel). 2020 Jun 24;9(6):789. doi: 10.3390/plants9060789 (PMC7357122; doi:10.3390/plants9060789)
Supplement: Supplementary file 1 [file plants-09-00789-s001.pdf]

Article

Phytochemical Analysis and Establishment of Embryogenic Cell Suspension and *Agrobacterium*-mediated Transformation for Farmer Preferred Cultivars of West African Plantain (*Musa* spp.)

Supplementary Materials: The following are available online at www.mdpi.com/xxx/s1, Figure S1: Transient expression of *gusA* gene across the various treatments and co-cultivation periods of *Agrobacterium*-mediated transformation of different plantain cultivars; Agbagba, Obino l'Ewai, and Orishele, Table S1: Various components of reaction for determination of total antioxidants, Table S2: Components of reaction mix for determination of total phenolics, Table S3: Reaction mixture for determination of total flavonoids, Table S4: Reaction mixture for determination of Tannin content.

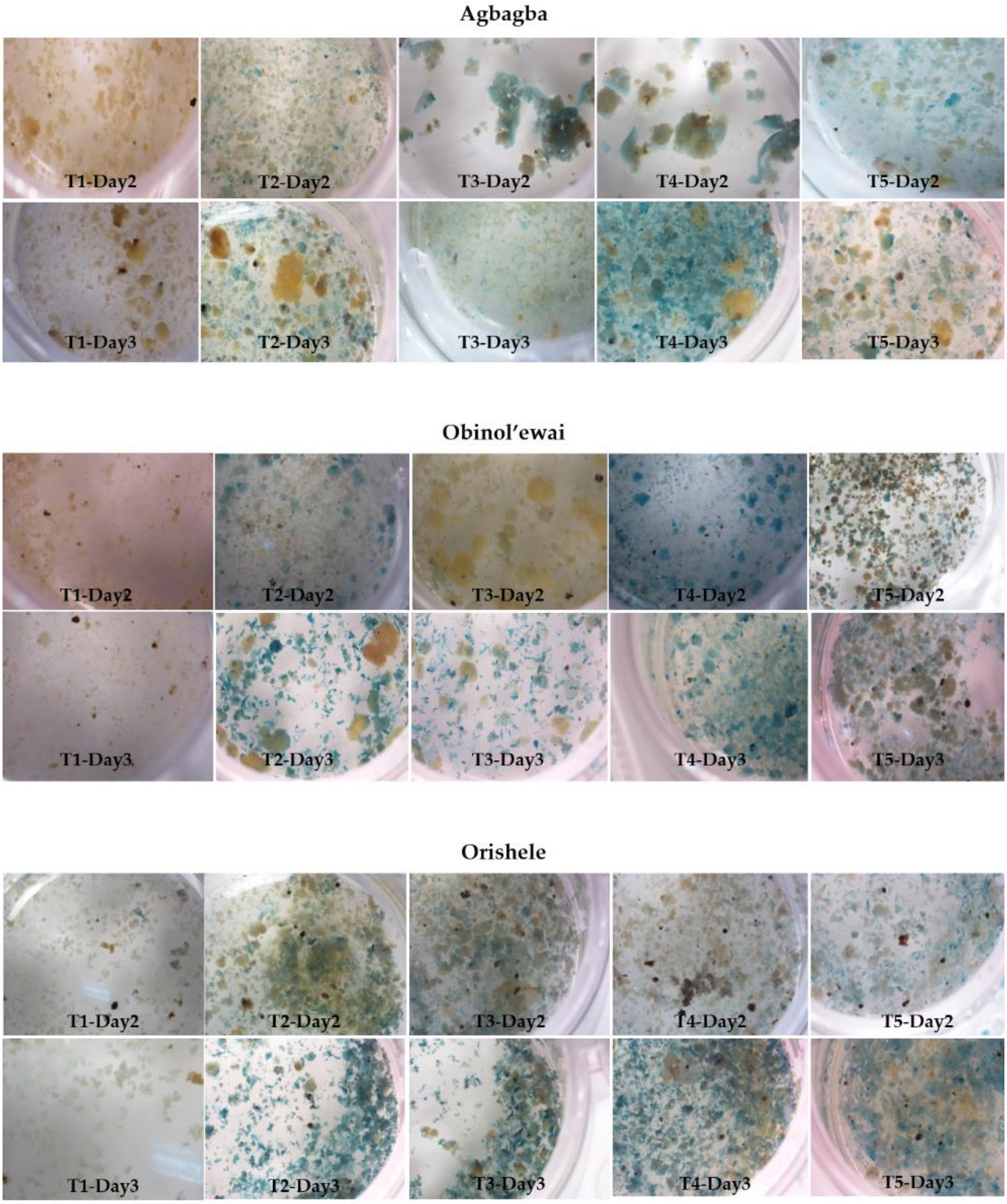

Figure S1: Transient expression of *gusA* gene across the various treatments and co-cultivation periods of *Agrobacterium*-mediated transformation of different plantain cultivars; Agbagba, Obino l'Ewai and Orishele.

Table S1: Various components of reaction mix for determination of total antioxidants.

Sample ID	Concentration of Gallic acid calibration standards (µg)/ml	Volume of calibration standards (µl)	Volume of 60mM DPPH (µl)
C-000(Methanol)	0	50	50
C-001	5	50	50
C-002	10	50	50
C-003	20	50	50
C-004	30	50	50
C-005	40	50	50
C-006	50	50	50
Sample	-	50	50

Table S2: Components of reaction mix for determination of total phenolics.

Sample ID	Concentration of Gallic acid calibration standards (µg)/ml	Volume of caliberation standards (µl)	Volume of Folin-Ciocalteu Phenol solution, 0.2 N (µl)	Volume of Na_2CO_3, 7% (µl)
C-000(Methanol)	0	20	100	80
C-001	10	20	100	80
C-002	20	20	100	80
C-003	40	20	100	80
C-004	60	20	100	80
C-005	80	20	100	80
C-006	100	20	100	80
Sample		20	100	80

Table S3: Reaction mixture for determination of total flavonoids.

Sample ID	Concentration of catechin acid calibration standards (μg)/ml	Volume of calibration standards (μl)	Volume of water(μl)	Volume of 5 % $NaNO_2$ (μl)	Volume of 10 % $AlCl_3$ (μl)	Volume of 2M NaOH (μl)
C-000(Methanol)	0	20	80	10	10	80
C-001	10	20	80	10	10	80
C-002	20	20	80	10	10	80
C-003	40	20	80	10	10	80
C-004	60	20	80	10	10	80
C-005	80	20	80	10	10	80
C-006	100	20	80	10	10	80
Sample		20	80	10	10	80

Table S4. Reaction mixture for determination of Tannin content.

Sample ID	Concentration of Tannic acid calibration standards (μg)/ml	Volume of calibration standards (μl)	Volume of Folin-Denis solution, 20% (μl)	Volume of Na_2CO_3, 7% (μl)
C-001(Methanol)	0	50	50	100
C-002	20	50	50	100
C-003	40	50	50	100
C-004	60	50	50	100
C-005	80	50	50	100
C-006	100	50	50	100
Sample		50	50	100
